# Restriction fragment length polymorphism of the L-*myc* gene is not a prognostic factor in bladder cancer patients

**DOI:** 10.1038/sj.bjc.6690295

**Published:** 1999-04

**Authors:** M J Ejarque, M Vicente, M Bernués, A Oliver, J Vicente, G Capellá, F Lluís, G Chéchile

**Affiliations:** Urology Service, Laboratory Service, Fundació Puigvert, Barcelona, Spain; Institut de Biologia Fonamental, Universitat Autònoma de Barcelona, Bellaterra, Barcelona, Spain; Laboratori d'Investigació Gastrointestinal, Hospital de la Santa Creu i Sant Pau, Barcelona, Spain

**Keywords:** proto-oncogenes, restriction fragment length polymorphism, bladder cancer, prognostic factors

## Abstract

The L-*myc* restriction fragment length polymorphism has been suggested to be of prognostic significance in some types of primary tumours. We examined the prognostic and susceptibility significance of the L-*myc* genotype in a group of 98 bladder cancer patients. The L-*myc* genotype did not correlate with any pathologic parameter and does not offer any clinical utility in patients with bladder cancer. © 1999 Cancer Research Campaign


*British Journal of Cancer* (1999) **79**(11/12), 1855–1858
© 1999 Cancer Research Campaign
Article no. bjoc.1998.0295

Short communication

### Restriction fragment length polymorphism of the L-*myc* gene is not a prognostic factor in bladder cancer patients

**MJ Ejarque[1], M Vicente[1], M Bernués[3], A Oliver[2], J Vicente[1], G Capellá[4], F Lluís[4] and G Chéchile[1]**

[1]Urology Service and [2]Laboratory Service, Fundació Puigvert, Barcelona, Spain; [3]Institut de Biologia Fonamental, Universitat Autònoma de Barcelona, Bellaterra, Barcelona, Spain; [4]Laboratori d'Investigació Gastrointestinal, Hospital de la Santa Creu i Sant Pau, Barcelona, Spain

**Summary** The L-*myc* restriction fragment length polymorphism has been suggested to be of prognostic significance in some types of primary tumours. We examined the prognostic and susceptibility significance of the L-*myc* genotype in a group of 98 bladder cancer patients. The L-*myc* genotype did not correlate with any pathologic parameter and does not offer any clinical utility in patients with bladder cancer.

**Keywords:** proto-oncogenes; restriction fragment length polymorphism; bladder cancer; prognostic factors

The restriction enzyme Eco RI identifies a two-allele system for the L-*myc* gene with fragment sizes of 10.0 and 6.6 kilobases and results in three possible allelotypes: homozygotic for the large allele (LL), homozygotic for the small allele (SS) or heterozygotic (LS). The prognostic significance of the L-*myc* allelotype of genomic DNA has been studied previously in several tumours (lung, colorectal, breast, soft tissue, leukaemia, lymphoma, stomach, kidney, oral cavity and liver). However, conficting results were reported. In this study we have investigated the L-*myc* genotype of 98 primary bladder cancer patients in an attempt to determine its utility as a prognostic, and as a susceptibility, factor in a Catalonian population.

### MATERIALS AND METHODS

Ninety-eight patients (88 males, 10 females, aged 38–91), diagnosed at Fundació Puigvert as having primary bladder cancer, were studied for L-*myc* restriction fragment length polymorphism (RFL); P 53 were superficial bladder tumours and 45 were invasive tumours. In addition, 110 normal DNA specimens from healthy blood donors (66 males, 44 females, aged 2–81) were analysed to investigate the genotype frequency of L-*myc* proto-oncogene.

High molecular weight DNA was isolated by standard phenol–chloroform methods. Restriction digests of DNA were performed with endonuclease Eco RI under standard conditions, electrophoresed in a 0.8% agarose gel, denaturated, transferred to Hybond® nylon membrane (Amersham) and hybridized with a $^{32}$P-labelled probe by the method essentially described by Southern (1975).

*Received 4 June 1998*
*Revised 13 August 1998*
*Accepted 21 August 1998*

*Correspondence to:* G Chéchile, Cartagena 340, 08025, Barcelona, Spain

All results obtained in these studies were analysed for statistical significance by the $\chi^2$ test with Yates' correction for adjustment when necessary. Differences between the two populations were judged significant at $P < 0.05$. Kaplan–Meier curves were constructed to analyse patient survival between different genotypes (log-rank test).

### RESULTS

Eco RI-digested DNA probed with the L-*myc* probe results in two fragments of 10 kb (L) and 6.6 kb (S), which are due to an Eco RI restriction site polymorphism (Figure 1). The distribution of the three genotypes (LL, LS, SS) in the control and patient groups is shown in Table 1. Chi-squared analysis showed that there was no difference between the distribution of the three genotypes of patient group and controls, and all are in accordance with Hardy–Weinberg equilibrium.

When the patients in the current study were examined for a relationship between the presence of S-alleles (LS or SS individuals) and distant metastasis, no association was found ($P = 0.51$). Nor was there a significant association between homozygosity for the

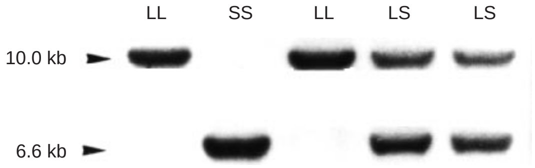

**Figure 1** Examples of Southern hybridization analysis of the L-*myc* gene. DNA samples obtained from peripheral leucocytes of five control individuals are shown. Genotypes of lanes 1 and 3 were LL, that of lane 2 was LS, and those of lanes 4 and 5 were SS

**Table 1** Distribution of L-*myc* genotypes and allele frequencies in patients and normal controls

| | Genotypes | | | | | | Allele frequency | | |
|---|---|---|---|---|---|---|---|---|---|
| | LL | LS | SS | Total | $\chi^2$ | (*P*) | L | S | (*P*) |
| Controls | 40 | 45 | 25 | 110 | | | 0.568 | 0.432 | |
| Patients | 32 | 45 | 21 | 98 | 0.546 | > 0.05 | 0.556 | 0.444 | > 0.05 |

**Table 2** Data on patients with bladder cancer and the distribution of the L-*myc* genotypes

| | | DNA pattern of L-*myc* no. of cases | | |
|---|---|---|---|---|
| **Total number** | **Total** | **LL** | **LS** | **SS** |
| Age (years) | | | | |
| < 60 | 10 | 3 | 6 | 1 |
| 61–70 | 37 | 16 | 16 | 5 |
| > 70 | 51 | 14 | 26 | 11 |
| Sex | | | | |
| Male | 88 | 28 | 44 | 16 |
| Female | 10 | 4 | 1 | 5 |
| Smoking | | | | |
| Yes | 71 | 23 | 34 | 14 |
| No | 27 | 9 | 11 | 7 |
| Grade of differentiation | | | | |
| G1 + G2 | 45 | 14 | 19 | 12 |
| G3 + anaplas. + indifferentiated | 53 | 18 | 26 | 9 |
| TNM stages | | | | |
| Ta + T1 | 53 | 18 | 25 | 10 |
| T2 + T3 + T4 | 45 | 14 | 20 | 11 |
| Recurrence[a] | | | | |
| R + | 46 | 11 | 26 | 9 |
| R – | 52 | 21 | 19 | 12 |
| Distant metastasis[b] | | | | |
| M + | 28 | 7 | 15 | 6 |
| M – | 68 | 25 | 29 | 14 |
| Nodal metastasis[c] | | | | |
| N + | 15 | 4 | 9 | 2 |
| N – | 71 | 26 | 30 | 15 |
| Urethral involvement | | | | |
| U + | 5 | – | 3 | 2 |
| U – | 93 | 32 | 42 | 19 |
| Prostate involvement[d] | | | | |
| P + | 9 | 2 | 5 | 2 |
| P – | 79 | 26 | 39 | 14 |

[a]Number of patients with previous relapses (R+) versus number of patients without previous relapses (R–). [b]This variable was not possible to examine in two cases. [c]This variable was not possible to examine in 12 cases. [d]Ten female patients were excluded of this examination.

S-allele and nodal metastasis ($P$ = 0.46). Further analysis revealed no significant associations with grade of differentiation of tumours, TNM stages, number of previous and subsequent relapses, and urethral and prostate involvement (Table 2). No association was observed between L-*myc* RFLP and survival of bladder cancer patients (Figure 2).

### DISCUSSION

An Eco RI polymorphism of the L-*myc* proto-oncogene was reported with the initial description of the gene (Nau et al, 1985) and the site of the polymorphism subsequently located in the second intron (Kaye et al, 1988). Studies of this polymorphism have linked it to both cancer susceptibility and prognosis. The role of L-*myc* in cancer susceptibility has been implied by studies showing differences in genotype frequencies between cancer patients and controls (Kato et al, 1990; Dolcetti et al, 1991; Crossen et al, 1994). The role of L-*myc* in prognosis has been suggested by studies showing that, among cancer patients, those that carry an S-allele (either LS or SS genotype) have earlier lymph node involvement or metastasis, or poorer survival than those who have genotype LL (Kakehi and Yoshida, 1989; Champeme et al, 1992; Kawashima et al, 1992). In contrast, Taylor et al (1993) reported a protective effect for the SS genotype in hepatocellular carcinoma. Hence, the association of the L-*myc* genotype with susceptibility and prognosis appears to vary with tumour type.

 *© Cancer Research Campaign 1999*

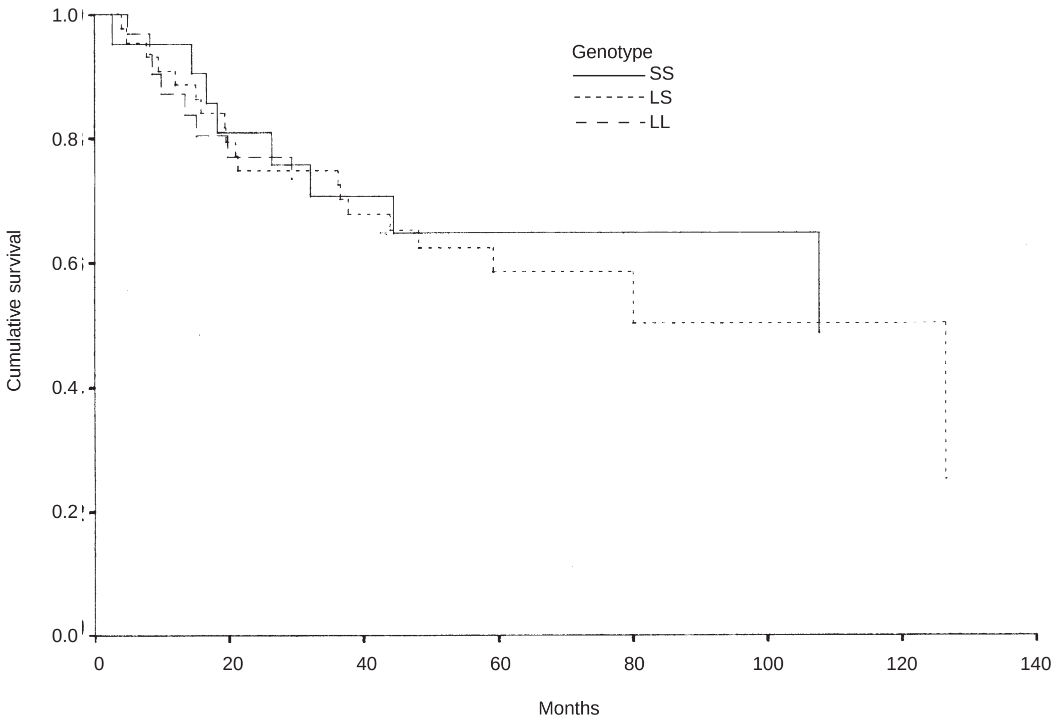

**Figure 2** Survival curves of bladder cancer patients with LL, LS and SS L-*myc* RFLP (log-rank test, $P = 0.53$)

In this study, we examined the relationship between the L-*myc* RFLP pattern and the clinical features of bladder cancer in 98 cases. A preponderance of the LL, LS or SS fragments was not observed in the bladder cancer patients compared to normal healthy individuals; thus, presence of a particular allele did not indicate predisposition to bladder cancer. Similar L-*myc* allelic frequencies were also found in previously reported analyses.

Our results fail to support the hypothesis that the L-*myc* locus is involved in a genetic predisposition to bladder cancer. These results confirm those of Ikeda et al in colorectal cancer (1988), Tefre et al in lung cancer in Norway (1990), Ishizaki et al (1990) and Champeme et al (1992) in breast cancer, Saranath et al (1990) in oral cancer, Weston et al (1992) in lung cancer in the USA, Mironov et al (1994) in gastric cancer in Russia and Presti et al (1996) in renal cancer. On the other hand L-*myc* RFLP is correlated with the extent of metastasis of lung cancer in Japan (Kawashima et al, 1988, 1992) and in renal cancer (Kakehi and Yoshida, 1989); Ishizaki et al (1990) also reported that the presence of the S-allele is associated with poor prognosis due to metastasic lesion in gastric cancer. Kato et al (1990) proposed that Japanese men with the S-allele may be prone to development of osteosarcoma and Saranath et al (1990) observed that patients with oral cancer with a genotype including an S fragment are more likely to develop a poorly to moderately differentiated tumour, or a larger tumour, than patients without an S fragment.

Our results show that the L-*myc* genotype does not correlate with tumour grade or stage in patients with bladder carcinoma. No significant correlation was observed between the L-*myc* genotype and the presence of nodal metastases or distant metastases at the time of surgery or with subsequent disease relapse.

This study did not demonstrate an association between the L-*myc* genotype and disease status in bladder cancer. It seems that L-*myc* polymorphism is not suitable as a prognostic and susceptibility marker in the bladder cancer patients studied.

### ACKNOWLEDGEMENTS

This work was partially suported by a grant from Ministerio de Educación y Ciencia (DGICT PM 910123). We thank Japanese Research Bank for the L-*myc* probe.

### REFERENCES

Champeme MH, Bieche Y, Latil A, Hacene K and Liderau R (1992) Association between restriction fragment length polymorphism of the L-*myc* gene and lung metastasis in human breast cancer. *Int J Cancer* **50**: 6–9

Crossen PE, Morrison MJ and Colls BM (1994) Increased frequency of the S allele of the L-*myc* oncogene in non-Hodgkin's lymphoma. *Br J Cancer* **69**: 759–761

Dolcetti R, Pelucchi S, Maestro R, Rizzo S, Pastore A and Boiocchi M (1991) Proto-oncogene allelic variations in human squamous cell carcinomas of the larynx. *Eur Arch Otorhinolaryngol* **248**: 279–285

Ikeda Y, Ishizaka Y, Ochiai M, Sakai R, Itabashi M, Honda M, Sugimura T and Nagao M (1988) No correlation between L-*myc* restriction fragment length polymorphism and malignancy of human colorectal cancers. *Jpn J Cancer Res (Gann)* **79**: 674–676

Ishizaki K, Kato M, Ikenaga M, Honda K, Ozawa K and Toguchida J (1990) Correlation of L-*myc* genotypes to metastasis of gastric and breast cancer. *Natl Cancer Inst* **82**: 238–239

Kakehi Y and Yoshida O (1989) Restriction fragment length polymorphism of the L-*myc* gene and susceptibility to metastasis in renal cancer patients. *Int J Cancer* **43**: 391–394

Kato M, Toguchida J, Honda K, Sasaki MS, Ikenaga M, Sugimoto M, Yamaguchi T, Kotoura Y, Yamamuro T and Ishizaki K (1990) Elevated frequency of a specific allele of the L-*myc* gene in male patients with bone and soft-tissue sarcomas. *Int J Cancer* **45**: 47–49

Kawashima A, Shikama H, Imoto K, Izawa M, Naruke T, Okabayashi E and Nishimura S (1988) Close correlation between restriction fragment length polymorphism of the L-*myc* gene and metastasis of human lung cancer to the lymph nodes and others organs. *Proc Natl Acad Sci USA* **85**: 2353–2356

Kawashima K, Nomura S, Hirai H, Fukushi S, Karube T, Takeuchi K, Naruke T and Nishimura S (1992) Correlation of L-*myc* RFLP with metastasis, prognosis and multiple cancer in lung-cancer patients. *Int J Cancer* **50**: 557–561

Kaye F, Battey J, Nau M, Brooks B, Seifter E, DeGreve J, Birrer M, Sausville E and Minna J (1988) Structure and expression of the human L-*myc* gene reveal a complex pattern of alternative mRNA processing. *Mol Cell Biol* **8**: 186–195

Mironov NM, Aguelon AM, Potapova GI, Gorbunov OV, Klimenkov AA and Yamasaki H (1994) L-*myc* allele polymorphism and prognosis for metastases in Russian gastric cancer patients. *Eur J Cancer* **30**: 1732

Nau MM, Brooks BJ, Battey J, Sausville E, Gazdar AF, Kirsch IR, McBride OW, Bertness V, Hollis GF and Minna JD (1985) L-*myc*, a new *myc*-related gene amplified and expressed in human small lung cancer. *Nature* **318**: 69–73

Presti JC Jr, Hinckley J and Reuter VE (1996) L-*myc* allelotype in renal cell carcinoma. *Cancer Genet Cytogenet* **88**: 66–68

Saranath D, Panchal RG, Nair R, Metha AR, Sanghavi V and Deo MG (1990) Restriction fragment length polymorphism of the L-*myc* gene in oral cancer patients. *Br J Cancer* **61**: 530–533

Southern EM (1975) Detection of specific sequences among DNA fragments separated by gel electrophoresis. *J Mol Biol* **98**: 503–517

Taylor JA, Bell DA and Nagorney D (1993) L-*myc* proto-oncogene alleles and susceptibility to hepatocellular carcinoma. *Int J Cancer* **54**: 927–930

Tefre T, Borresen AL, Aamdal S and Brogger A (1990) Studies of the L-*myc* DNA polymorphism and relation to metastasis in Norwegian lung cancer patients. *Br J Cancer* **61**: 809–812

Weston A, Caporaso EN, Perrin LS, Sugimura H, Tamai S, Krontiris TG, Trump BF, Hoover RN and Harris CC (1992) Relationship of H-*ras*-1, L-*myc* and *p53* polymorphisms with lung cancer risk and prognosis. *Environ Health Perspect* **98**: 61–67

 *© Cancer Research Campaign 1999*

